# Attitudes toward COVID-19 Vaccination: A Survey of Chinese Patients with Rheumatic Diseases

**DOI:** 10.3390/vaccines10101604

**Published:** 2022-09-23

**Authors:** Zixi Yi, Zhongqiang Yao, Dan Xu, Chuanhui Xu, Wenqiang Fang, Zhanfei Guo, Yong Wang, Jianlin Huang, Qin Li, Hong Zhang, Anbin Huang, Lijun Wu, Zhenbiao Wu, Huifang Guo, Fengxiao Zhang, Jing Lu, Zhenchun Zhang, Zhongming Yu, Zhanyun Da, Li Luo, Bin Wu, Henglian Wu, Lin Zeng, Rong Mu

**Affiliations:** 1 Department of Rheumatology and Immunology, Peking University Third Hospital, Beijing 100191, China; 2 Department of Rheumatology, Allergy and Immunology, Tan Tock Seng Hospital, Singapore 999002, Singapore; 3 Department of Rheumatology and Immunology, Xinxiang Central Hospital, Xinxiang 453000, China; 4 Department of Rheumatology and Immunology, The Southwest Hospital of Army Medical University, Chongqing 404100, China; 5 Department of Rheumatology and Immunology, Sun Yat-sen University Sixth Affiliated Hospital, Guangzhou 510275, China; 6 Department of Rheumatology and Immunology, The First People’s Hospital of Yunnan Province, Kunming 650300, China; 7 Department of Rheumatology and Immunology, Wuhan Union Hospital, Tongji Medical College, Huazhong University of Science and Technology, Wuhan 430074, China; 8 Department of Rheumatology and Immunology, People’s Hospital of Xinjiang Uygur Autonomous Region, Urumqi 830001, China; 9 Department of Rheumatology and Immunology, Air Force Medical University, Xi’an 710000, China; 10 Department of Rheumatology and Immunology, The Second Hospital of Hebei Medical University, Shijiazhuang 050000, China; 11 Department of Rheumatology and Immunology, Hebei General Hospital, Shijiazhuang 050000, China; 12 Department of Rheumatology and Immunology, The First Hospital of China Medical University, Shenyang 110000, China; 13 Department of Rheumatology and Immunology, Linyi People’s Hospital, Linyi 273300, China; 14 Department of Rheumatology and Immunology, Shaoxing People’s Hospital, Shaoxing 310009, China; 15 Department of Rheumatology and Immunology, Affiliated Hospital of Nantong University, Nantong 226001, China; 16 Department of Rheumatology and Immunology, Xinjiang Medical University Affiliated First Hospital, Urumqi 830001, China; 17 Department of Rheumatology and Immunology, First People’s Hospital of Jingzhou, Jingzhou 434000, China; 18 Department of Rheumatology and Immunology, Dongguan Tungwah Hospital, Dongguan 523000, China; 19 Research Center of Clinical Epidemiology, Peking University Third Hospital, Beijing 100191, China

**Keywords:** rheumatic diseases, COVID-19, attitudes, vaccine

## Abstract

The coronavirus disease 2019 (COVID-19) pandemic has imposed enormous morbidity and mortality burdens. Patients with rheumatic diseases (RDs) are vulnerable to the COVID-19 infection, given their immunocompromised status. Ensuring acceptance of the COVID-19 vaccine is important and has attracted attention by health professionals. In this study, we designed an online cross-sectional survey that used an online questionnaire from 8 May 2021 to 4 October 2021. Attitudes toward the COVID-19 vaccination, personal information, current disease activity status, adverse events (AEs), and knowledge sources of vaccines were collected. Descriptive statistics, nonparametric tests, and ordinal logistic regression were used to analyze the data. A total of 1022 questionnaires were received, among which 70.2% (720/1022) of patients with RDs agreed to vaccination, while only 31.6% of patients were actually vaccinated. Male, employed, high-income patients and those with inactive disease showed a more positive attitude. Concerns of AEs and disease flare were the main factors affecting vaccination willingness. Only 29.6% (304/1022) of patients thought they had received enough information about the COVID-19 vaccine from their doctors. In conclusion, most patients with RDs in China intended to get vaccinated, although the vaccination rate in this particular population was low. Rheumatologists should take more responsibility in COVID-19 vaccination education of patients with RDs.

## 1. Introduction

Globally, the coronavirus disease 2019 (COVID-19) pandemic has caused 601,189,435 confirmed cases, with 6,475,346 deaths as of 2 September 2022 [1], which has imposed enormous morbidity and mortality burdens. Patients with rheumatic diseases (RDs) are at a high risk for COVID-19 due to their immunocompromised status [2]. To control the mortality of COVID-19, vaccines with different targets and vectors have been developed. The efficacy and safety of COVID-19 vaccines have been widely accepted by health professionals [3,4,5,6,7,8,9]. Ensuring acceptance of the COVID-19 vaccine by patients is just as important. However, people’s attitudes towards the COVID-19 vaccine remain unclear, especially in patients with RDs.

There are a few studies that focus on attitudes towards the COVID-19 vaccine among patients with RDs [10,11,12,13,14,15,16] and show that the acceptance or willingness of COVID-19 vaccination varies greatly in different countries and cultural backgrounds, which ranged from 29.2% in Turkey [15] to 65% in Australia [16]. The influence of age, education standards, and disease status had been mixed. In addition, previous studies showed that the reasons for refusal were concerns of adverse events (AEs), disease flare, or distrust in the COVID-19 vaccine [10,11,12,13,14,15]. Although the percentages of reported AEs were different, no fatal AEs, significant safety concerns, or severe disease flares were identified in these studies after COVID-19 vaccinations in patients with RDs. Inactivated vaccines and mRNA COVID-19 vaccines have demonstrated a favorable safety profile in patients with RDs [4,5,6]. Most rheumatologists have also reached an agreement that nearly all patients with RDs should be recommended for vaccination against COVID-19 [17,18,19,20]. It remains unclear how well patients with RDs know this information. In addition, most studies only reported the acceptance or willingness for vaccination against COVID-19 but did not report the role of attitudes [10,11,12,13,14,15,16]. Reported intentions may not always translate to the use of vaccines. Therefore, the objectives of this study were to investigate the attitudes of patients with RDs toward vaccination against COVID-19 and analyze the underlying related factors.

## 2. Materials and Methods

### 2.1. Participants and Survey Administration

We conducted a cross-sectional, anonymous, multi-center survey using an online questionnaire from 8 May 2021 to 4 October 2021. Patients with RDs were recruited from the tertiary hospitals in the cities of Beijing, Tianjin, and Shanghai as well as the provinces of Henan, Chongqing, Guangzhou, Yunnan, Wuhan, Shanxi, Hebei, Liaoning, Shandong, Inner Mongolia, Zhejiang, Jiangsu, Jiangxi, Jilin, Hunan, Heilongjiang, Xinjiang, Sichuan, Shanxi, Guangzhou, Gansu, Fujian, Anhui, and Hubei. After informed consent, they were invited to answer the questionnaires online with the WeChat app. For those who had difficulties using smart phones, family members helped filling in the questionnaire. The formal questionnaire is detailed in the Supplementary Materials.

### 2.2. Measures

This survey included 31 items that were compiled through a review of references and consultations with experts, which consisted of questions concerning personal information, attitudes toward the COVID-19 vaccination, status of vaccination, AEs, and the sources of knowledge about the COVID-19 vaccine (Supplementary Materials, Table S1). The personal information included age, gender, income, education levels, and employment status. Age and income could be left blank due to privacy.

### 2.3. Attitudes toward COVID-19 Vaccination

Patients were asked to score their attitudes toward the COVID-19 vaccination on a 5-point Likert scale (5 = completely agree, 4 = somewhat agree, 3 = neutral/no opinion, 2 = somewhat disagree, and 1 = completely disagree). Patients with scores ≤ 3 were invited to explain the reasons for refusing vaccination, such as AEs, disease relapse, COVID-19 infection, and invalidity of vaccines. The results were also shown on a 5-point Likert scale. In addition, all patients were asked if they had consulted a rheumatologist about vaccination issues and a prediction of contracting a COVID-19 infection in the next 6 months. We investigated the impact of these factors on attitudes toward the COVID-19 vaccination.

### 2.4. Current Status of Vaccination and Adverse Events

We further surveyed the vaccination status of the patients and the influence of job requirements, community requirements, personal willingness, and doctor’s advice on vaccination. Vaccinated patients were asked to report AEs after vaccination in an open question format. Clear causality was not limited here, and any discomfort after vaccination could be reported.

### 2.5. Knowledge Sources of the COVID-19 Vaccine

We investigated the sources of knowledge regarding the COVID-19 vaccine, including the Internet, television, science popularization, discussion with colleagues or friends, propaganda of the community, or guidance of doctors.

### 2.6. Statistical Analysis

The statistical analyses were performed by the Statistical Product Service Solutions 27.0. We used frequencies and percentages to summarize the participants’ characteristics. Then, an ordinal logistic regression analysis was used to analyze the variables of personal information influencing the attitudes towards the COVID-19 vaccine (*p* < 0.05). Owing to the small sample size, we merged the data of “somewhat disagree” and “completely disagree” in the statistical analysis; attitudes toward COVID-19 were divided into four orderly groups: completely agree (score = 4), somewhat agree (score = 3), neutral (score = 2), and somewhat or completely disagree (score = 1). A test of parallel lines checked the proportional odds assumption that the odds ratios (OR) for each predictor were similar. The OR and 95% confidence intervals (95% CI) were calculated for each variable. Because there were some missing data in age and income, the acquired data of age and income were analyzed respectively. To find the decisive reasons for vaccination or refusal, the related samples were analyzed with the Friedman’s two-way analysis of variance by ranks summary and the Wilcoxon signed rank test summary (a two-sided test with *p* < 0.05 was considered statistically significant).

## 3. Results

### 3.1. Demographic Characteristics of Participants

The demographic characteristics of the patients are shown in Table 1. A total of 20% (203/1022) of the patients were male, and 80% (819/1022) were female. A total of 14 (1.4%) patients declined to disclose their age, and 54 (5.4%) patients declined to disclose their income. The median (SD) age of participants was 39 (12.5) years. Most of the patients were in inactive disease (81.3%, 814/1022). Rheumatoid arthritis (28.0%, 287/1022) and systemic lupus erythematosus (23.1% 237/1022) are dominant in the participants.

### 3.2. Attitudes toward COVID-19 Vaccination

**Figure 1:**
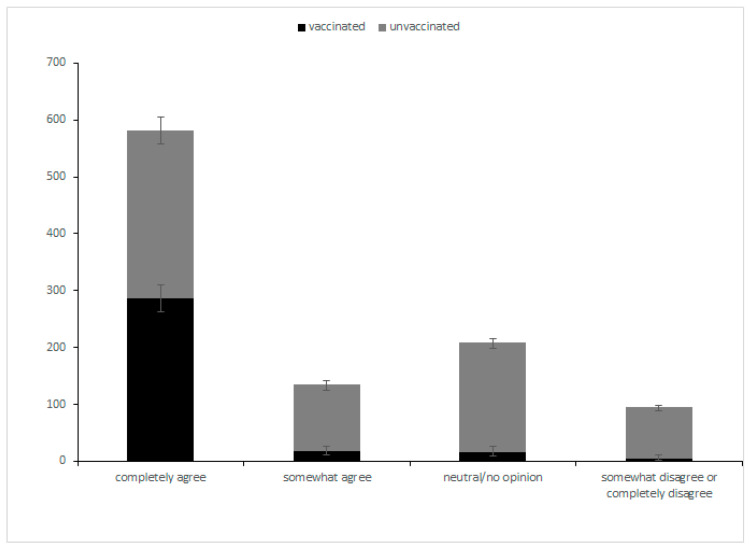
Attitudes toward the COVID-19 vaccination and the vaccination rate.

Attitudes toward the COVID-19 vaccination are shown in Figure 1. A total of 57.0% (582/1022) (95% CI, 53.8–60.0%) completely agreed with vaccination; 13.2% (135/1022) (95% CI, 11.2–15.5%) somewhat agreed, and 20.5% (209/1022) (95% CI, 18.0–23.1%) are neutral. Only 9.4% (96/1022) (95% CI, 7.7–11.4%) somewhat or completely disagreed with vaccination. An ordinal logistic regression analysis (Table 2) showed that male, employed, high-income patients, with an inactive disease status and a doctor consultation about vaccination recommendation, were statistically significant factors for a positive attitude towards the COVID-19 vaccination (*p* < 0.05).

We merged the data of “somewhat disagree” and “completely disagree” because of the small sample size. The black part represents the proportion of patients who were vaccinated, while the grey part represents the unvaccinated.

The patients who did not support vaccination (neutral, somewhat disagree, and completely disagree, *n* = 306) rated the reasons on a 5-point Likert scale, including concerns about AEs, disease relapse, COVID-19 infection, and invalidity of the vaccines. Table 3 and Figure S1 present the proportion of every score for different reasons. These reasons were compared with each other, and the results of The Related-Samples Wilcoxon Signed Rank Test Summary showed that concerns about disease flare (*p* < 0.01) were the main reasons for refusing vaccination, and fear of AEs ranked second (*p* < 0.01). Worry over the vaccine causing a COVID-19 infection or invalidity of the vaccines were not the main reasons for a negative attitude toward the COVID-19 vaccine.

### 3.3. Knowledge of the COVID-19 Vaccination

A total 69.8% (716/1022) of the patients acquired knowledge about the COVID-19 vaccination from the Internet. The proportion of other sources was approximately 30% and included television, science popularization, discussion with colleagues or friends, propaganda of the community, and guidance of doctors (Table S2). Only 0.02% (2/1022) of the patients did not have any knowledge about the vaccination.

A total of 59.2% (607/1022) of the patients had consulted rheumatologists about the vaccination, and 60.7% of them (370/607) were recommended for vaccination, while only 41.52% (252/607) of the patients thought they acquired knowledge from doctors.

### 3.4. Confidence in the Pandemic

Most patients (87.9%, 898/1022) thought that they would not get a COVID-19 infection or that if they got COVID-19, the symptoms would be mild (7.1%, 73/1022). Only 5% (51/1022) of the patients viewed it as a serious condition. None of our enrolled RD patients already contracted the coronavirus (Table S2).

### 3.5. Current Status of Vaccination

**Figure 2:**
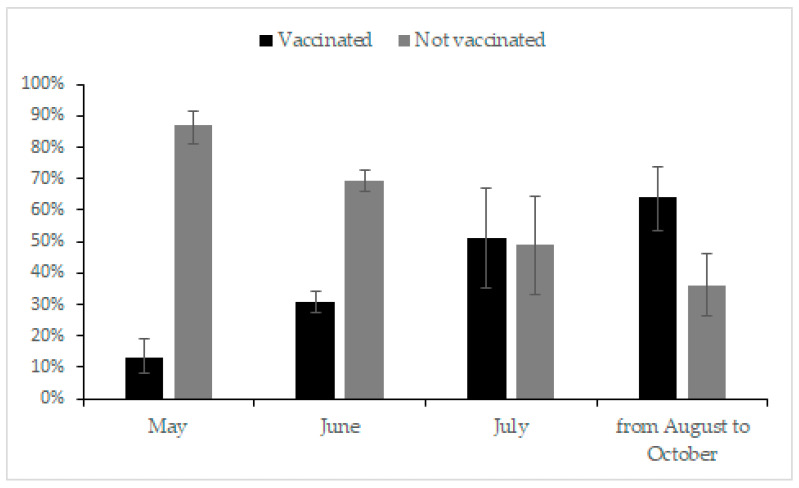
Vaccination rate of Chinese patients with rheumatic diseases in different time periods.

A total of 31.7% (324/1022, 95% CI, 28.9–34.7%) of patients with RDs were vaccinated. Of the 324 patients, 87.4% (283/324, 95% CI, 87.8–91.0%) were in inactive disease. The vaccination rate increased over time: 12.8% (21/164, 95% CI, 8.3–19.1%) in May, 30.6% (221/722, 95% CI, 27.3–34.1%) in June, 51.2% (21/41, 95% CI, 35.3–66.9%) in July, and 64.2% (61/95, 95% CI, 53.7–73.6%) from August to October (Figure 2). The vaccination rate in the “completely agree” subgroup was 49.1% (286/582, 95% CI, 45.0–53.3%), while the “somewhat agree”, “neutral/no opinion”, and “somewhat disagree/completely disagree” subgroups were 12.6% (17/135, 95% CI, 7.7–19.7%), 7.7% (16/209, 95% CI, 4.6–12.4%), and 5.2% (5/96, 95% CI, 1.9–12.3%), respectively (Figure 1).

The black part represents the proportion of patients who were vaccinated, while the grey part represents the unvaccinated. Because there were only a few patients in this study from August to October compared to other months, we consolidated the data in this part.

In order to explore which reason had the greatest impact on vaccinated patients, the patients who had been vaccinated (*n* = 324) rated the reasons for vaccination on a 5-point Likert scale (Table 3 and Figure S2). The related samples from the Wilcoxon signed rank test summary showed that personal willingness was the decisive reason for vaccination compared with work requirements, community requirements, and doctor’s recommendation (*p* < 0.001).

### 3.6. Adverse Events after Vaccination

A total of 231 of these patients (71.3%, 231/324) reported AEs after vaccination (Table 4), and only 0.5% (5/1022) patients reported disease flares. The most common AEs were redness, swelling, and pain at the inoculation site (39.9% (92/231)), followed by weakness (29.9% (69/231)) and myalgia (27.7% (64/231)).

## 4. Discussion

This is a study to investigate the attitudes towards vaccination against COVID-19, vaccination rate, and related factors in Chinese patients with RDs. The results of our survey indicated that most patients with RDs took a positive attitude toward vaccination. Further analysis showed that female, unemployed, low-income patients, and those with active disease had lower willingness. Future vaccination strategies should focus on these subpopulations. The overall vaccination rate was lower in patients with RDs in China than the whole population, and the personal willingness was the most important influencing factor of vaccination.

In our study, the inclination of vaccination in RDs patients was lower compared with the healthy people but in the forefront of RD patients in the world. When compared with healthy people, it is lower than that in healthy adults in China (91.3% [21] or 83.5% [22]), comparable to that in Italy (74.8% [23]) and America (68% [24]), and higher than that in Jordan, Kuwait, or other Arab countries (29.4–30.9% [25]) and Russia (41.7% [26]). In patients with RDs, the inclination to vaccinate is similar to that in Australia (65% [16]) and the Netherlands (61% [14]) and higher than that in Turkey (29.2% [15]). The subgroup of “completely agree” had a higher vaccination rate than other subgroups, and personal willingness is the decisive factor in patients who had, in fact, been vaccinated, which showed the role of attitudes towards the COVID-19 vaccine.

The vaccination rate of patients with RDs was lower than that of the whole Chinese population at the same time (31.7% vs. 78% as of 18 September 2021 [27]). Consistent with other studies, concerns of AEs and disease flare were the main factors affecting the willingness to vaccinate. However, as mentioned earlier, the COVID-19 vaccine has demonstrated a favorable safety profile and been recommended by experts [4,5,6,10,11,12,13,14,15,17,18,19,20]. One explanation is that our patients might know little about expert consensus from doctors. A consultation and vaccination recommendation from doctors significantly affected the attitude towards the COVID-19 vaccination. While our research showed that although nearly 60% of the patients consulted a rheumatologist, less than half of them thought they received enough knowledge about the COVID-19 vaccination from doctors, which implies that Chinese rheumatologists need to strengthen the communication with and education of the patients with RDs to increase vaccination rate and protect them in the COVID-19 pandemic.

We are aware of several limitations of this study. First, the survey spanned a long period of time. Thus, the rates may be varied over time under different social circumstances. However, from 8 May 2021 to 4 October 2021, there were only 5526 confirmed cases in the Chinese mainland (data from the official website of the National Health Commission), which implies the epidemic situation may not have had a heavy impact on our research results. Second, our research is a web-based questionnaire, so there was a risk of selection biases. Respondents with AEs would be more likely to take part in the survey, which may overestimate AEs. Third, our study lacked a contemporaneous control, so we utilized the data dispersed in published literature as a supplement.

## 5. Conclusions

In conclusion, most Chinese patients with RDs took a positive attitude towards the COVID-19 vaccination, and personal willingness was the most important factor affecting the vaccination rate. Many RDs patients are still reluctant to get vaccinated for fear of disease flare or AEs; thus, the vaccination status in this population was not satisfactory. Although vaccines have a good safety profile and most rheumatologists support that nearly all patients with RDs should be recommended for the COVID-19 vaccine, patients still do not have enough knowledge about the COVID-19 vaccination, and health professionals need to pay more attention to introducing information about the COVID-19 vaccination to patients with RDs.

## Figures and Tables

**Table 1:** Demographic data of the participants.

Characteristic of the Participants	Data (N = 1022)
Gender	
Male	203 (19.86%)
Female	819 (80.14%)
Age (y)	
18–24	68 (6.65%)
25–35	306 (29.94%)
36–55	514 (50.29%)
56–70	111 (10.86%)
>70	9 (0.88%)
Invalid Data	14 (1.37%)
Educational level	
Postgraduate degree or above	53 (5.19%)
Bachelor’s degree	263 (25.73%)
High school graduate or junior college	394 (38.55%)
Less than high school	316 (30.92%)
Monthly income (RMB)	
>10,000	71 (6.95%)
5000–10,000	189 (18.49%)
2500–5000	293 (28.67%)
<2500	418 (40.80%)
Did not report	54 (5.09%)
Employment status	
Employed	659 (64.48%)
Unemployed	363 (35.52%)
Location	
City	627 (61.35%)
Countryside	395 (38.65%)
Disease status	
Inactive disease	811 (79.35%)
Active disease	212 (20.65%)
Disease diagnosis	
RA	286 (27.98%)
SLE	236 (23.09%)
AS	171 (16.73%)
SSc	131 (12.82%)
SS	123 (12.04%)
Others	229 (22.41%)

RA = rheumatoid arthritis; SLE = systemic lupus erythematosus; AS = ankylosing spondylitis; SSc = systemic sclerosis/scleroderma; SS = Sjogren’s syndrome; others = other rheumatic diseases with a percentage under 5% in this survey, such as vasculitis, Behcet’s disease, IgG4-related disease, etc. A patient can suffer from more than two different rheumatic diseases.

**Table 2:** Logistic regression analysis of attitudes toward COVID-19 vaccines.

Factors	Parameter Estimates ^a^			
	OR	95% CI for OR		Sig.
		Lower Bound	Upper Bound	
Gender				
Male	1.65	1.2	2.28	0.002 *
Location				
Countryside	1.05	0.81	1.37	0.706
Employment status				
Unemployed	0.67	0.51	0.88	0.004 *
Disease status				
Active disease	0.5	0.37	0.66	0.000 **
Educational level (N = 1022)	0.97	0.83	1.14	0.741
Age (per 1 year) (N = 1010)	1	0.9	1.11	0.939
Monthly income (RMB) (N = 970)	1.29	1.13	1.47	0.000 **
Confidence (N = 1022)				
I will get seriously ill	0.57	0.34	0.96	0.022 *
I will get a mild case	1.29	0.79	2.08	0.302
I will not be infected	ref			
Have consulted a rheumatologist	1.68	1.33	2.14	0.000 **
Received knowledge and guidance from doctors				
	1.39	1.03	1.85	0.028 *
Rheumatologist recommend vaccination				
	3.53	2.53	4.91	0.000 **

* *p* < 0.05 ** *p* < 0.001. ^a^ Test of Parallel Lines, *p* value > 0.05.

**Table 3:** The reasons for vaccinating or refusing vaccination. 5=, 4=, 3=, 2= and 1=.

Reasons	5-Point Likert Scale				
	Completely Disagree (%)	or Somewhat Disagree (%)	Neutral/No Opinion (%)	Somewhat Agree (%)	Completely Agree (%)
Reasons for vaccinating (N = 324)					
Work requirements	66 (20.4)	9 (2.8)	30 (9.3)	19 (5.9)	200 (61.7)
Community requirements	70 (21.6)	19 (5.9)	28 (8.6)	29 (9.0)	178 (54.9)
Personal willingness	11 (3.4)	9 (2.8)	24 (7.4)	21 (6.5)	259 (79.9)
Doctor’s recommendation	55 (17.0)	20 (6.2)	35 (10.8)	37 (11.4)	177 (54.6)
Reasons for refusing (N = 305)					
Disease flare	41 (13.8)	15 (4.9)	32 (10.5)	27 (8.9)	190 (62.0)
Adverse effect	62 (20.7)	19 (6.2)	39 (12.8)	27 (8.9)	158 (51.5)
Causing COVID-19 infection	128 (43.0)	24 (7.9)	45 (14.8)	23 (7.5)	85 (27.5)
Invalidity of vaccines	139 (45.9)	31 (10.2)	46 (15.1)	22 (7.2)	67 (21.6)

**Table 4:** Adverse events after vaccination of patients with RDs ^a^.

Adverse Events	Date (N = 231)
Redness, swelling, and pain at the inoculation site	92 (39.83%)
Weakness	69 (29.87%)
Muscle soreness	64 (27.71%)
Headache	21 (9.09%)
Nausea	13 (5.63%)
Fever	12 (5.19%)
Flare	5 (2.16%)
Rash and itch	4 (1.73%)
Dizzy	2 (0.87%)
Thrombus	1 (0.43%)
Constipation	1 (0.43%)
Cold	1 (0.43%)

**^a^** A patient could suffer from more than two adverse events.

## Data Availability

The data presented in this study are available on request from the corresponding author. The data are not publicly available due to privacy.

## Supplementary Materials

The following supporting information can be downloaded at the following: https://www.mdpi.com/article/10.3390/vaccines10101604/s1, Figure S1: the related samples from the Wilcoxon signed rank test summary of reasons for refusing vaccination; Figure S2: the related samples from the Wilcoxon signed rank test summary of reasons for vaccination; Table S1: full questionnaire of attitudes toward COVID-19 vaccination; Table S2: knowledge source of COVID-19 vaccine and confidence of COVID-19 infection.
