# Supplementary material for: Attitudes toward COVID-19 Vaccination: A Survey of Chinese Patients with Rheumatic Diseases
Source: Vaccines (Basel). 2022 Sep 23;10(10):1604. doi: 10.3390/vaccines10101604 (PMC9611697; doi:10.3390/vaccines10101604)
Supplement: Supplementary file 1 [file vaccines-10-01604-s001.zip › vaccines-1925427-supplementary.pdf]

Table S1. Full questionnaire of Attitudes Toward COVID-19 Vaccination: A Survey in Chinese Patients with Rheumatic Diseases

Section A: Demegraphic information		
1	Gender	1. Male<br>2. Female
2	Age	___years old
3	Disease	1. Rheumatoid arthritis<br>2. Systemic lupus erythematosus<br>3. Sjogren's syndrome<br>4. Systemic sclerosis / scleroderma<br>5. Polymyositis and dermatomyositis<br>6. Ankylosing spondylitis<br>7. Psoriatic arthritis<br>8. Inflammatory bowel disease arthritis<br>9. Reactive arthritis<br>10. Adult still disease<br>11. Juvenile idiopathic arthritis<br>12. Vasculitis<br>13. Takayasu arteritis<br>14. Giant cell arteritis<br>15. Nodular polyarteritis<br>16. Behcet's disease<br>17. Osteoarthritis<br>18. Antiphospholipid syndrome<br>19. IgG4 related diseases<br>20. Rheumatic polymyalgia<br>21. Fibromyalgia syndrome<br>22. Undifferentiated connective tissue disease<br>23. Mixed connective tissue disease<br>24. other
4	Highest education level	1. Postgraduate degree or above<br>2. Bachelor's degree<br>3. High school graduate or junior college<br>4. Less than high school
5	Monthly income (RMB)	1. ＞10000<br>2. 5000-10000<br>3. 2500-5000<br>4. ＜2500<br>5. Inconvenient to disclose

6	Location	1. City<br>2. Countryside
7	Employment status	1. On employment<br>2. Not on employment
8	Disease status	1. Inactive disease<br>2. Active disease
9	Disease status	Inactive disease<br>Active disease

	**Section B: Attitudes toward COVID-19 vaccine**	
1	Rate your attitudes toward COVID-19 vaccine from 1-5	1. completely disagree<br>2. somewhat disagree<br>3. neutral/no opinion<br>4. somewhat agree<br>5. completely agree

	**Section C: The reasons for refusing vaccination**	
1	Rate your attitudes toward concerns about adverse effect from 1-5	1. completely disagree<br>2. somewhat disagree<br>3. neutral/no opinion<br>4. somewhat agree<br>5. completely agree
2	Rate your attitudes toward concerns about disease flare from 1-5	1. completely disagree<br>2. somewhat disagree<br>3. neutral/no opinion<br>4. somewhat agree<br>5. completely agree
3	Rate your attitudes toward concerns about causing COVID-19 infection from 1-5	1. completely disagree<br>2. somewhat disagree<br>3. neutral/no opinion<br>4. somewhat agree<br>5. completely agree
4	Rate your attitudes toward concerns about invalidity of vaccines from 1-5	1. completely disagree<br>2. somewhat disagree<br>3. neutral/no opinion<br>4. somewhat agree<br>5. completely agree

Section D: Current status of vaccination and adverse events		
1	Have you been vaccinated?	1. Yes<br>2. No
2	If you have been vaccinated, do you have any adverse effect after vaccination? (multiple choices, open questions)	1. redness, swelling and pain at the inoculation site<br>2. fever<br>3. weakness<br>4. nausea<br>5. headache<br>6. Muscle soreness<br>7. thrombus<br>8. Other____
3	If you have been vaccinated, rate the effect of personal willingness on vaccination from 1-5	1. completely disagree<br>2. somewhat disagree<br>3. neutral/no opinion<br>4. somewhat agree<br>5. completely agree
4	If you have been vaccinated, were you vaccinated because community requirements? Rate it from 1-5.	1. completely disagree<br>2. somewhat disagree<br>3. neutral/no opinion<br>4. somewhat agree<br>5. completely agree
5	If you have been vaccinated, were you vaccinated because work requirements? Rate it from 1-5.	1. completely disagree<br>2. somewhat disagree<br>3. neutral/no opinion<br>4. somewhat agree<br>5. completely agree
6	If you have been vaccinated, were you vaccinated because personal willingness? Rate it from 1-5.	1. completely disagree<br>2. somewhat disagree<br>3. neutral/no opinion<br>4. somewhat agree<br>5. completely agree
7	If you have been vaccinated, were you vaccinated because doctor's recommendation? Rate it from 1-5.	1. completely disagree<br>2. somewhat disagree<br>3. neutral/no opinion<br>4. somewhat agree<br>5. completely agree

Section E: Confidence of COVID-19 infection in future		
1	Do you think you will get COVID-19 in 6 months？	1. I will not be infected<br>2. I will get a mild case<br>3. I will get seriously ill<br>4. I have already had the coronavirus

Section F: Knowledge source of the COVID-19 vaccine.		
1	Where did you get COVID-19 vaccine knowledge?	1. Internet<br>2. Television<br>3. Popular science<br>4. Discussion with colleagues or friends<br>5. Propaganda of the community<br>6. Guidance of doctors<br>7. Do not understand any knowledge about vaccine
2	Have you ever consulted a rheumatologist?	1. Yes<br>2. No
3.	Did the rheumatologist suggest you get vaccinated?	1. Yes<br>2. No

Table S2. Knowledge source of COVID-19 vaccine and confidence of COVID-19 infection

Knowledge source and Confidence	Data	
Knowledge source*	N=1026	
Internet	716	(69.79%)
Television	316	(30.80%)
Popular science	354	(34.50%)
Discussion with colleagues or friends	267	(26.02%)
Propaganda of the community	366	(35.67%)
Guidance of doctors	304	(29.63%)
Do not understand any knowledge about vaccine	2	(0.19%)
Confidence	N=1026	
Do you think you will get COVID-19 in 6 months?		
I will not be infected	902	(87.91%)
I will get a mild case	73	(7.12%)
I will get seriously ill	51	(4.97%)
I have already had the coronavirus	0	(0.00%)

*A patient can choose more than two knowledge sources

Figure S1. The Related-Samples Wilcoxon Signed Rank Test Summary of reasons for refusing vaccination.

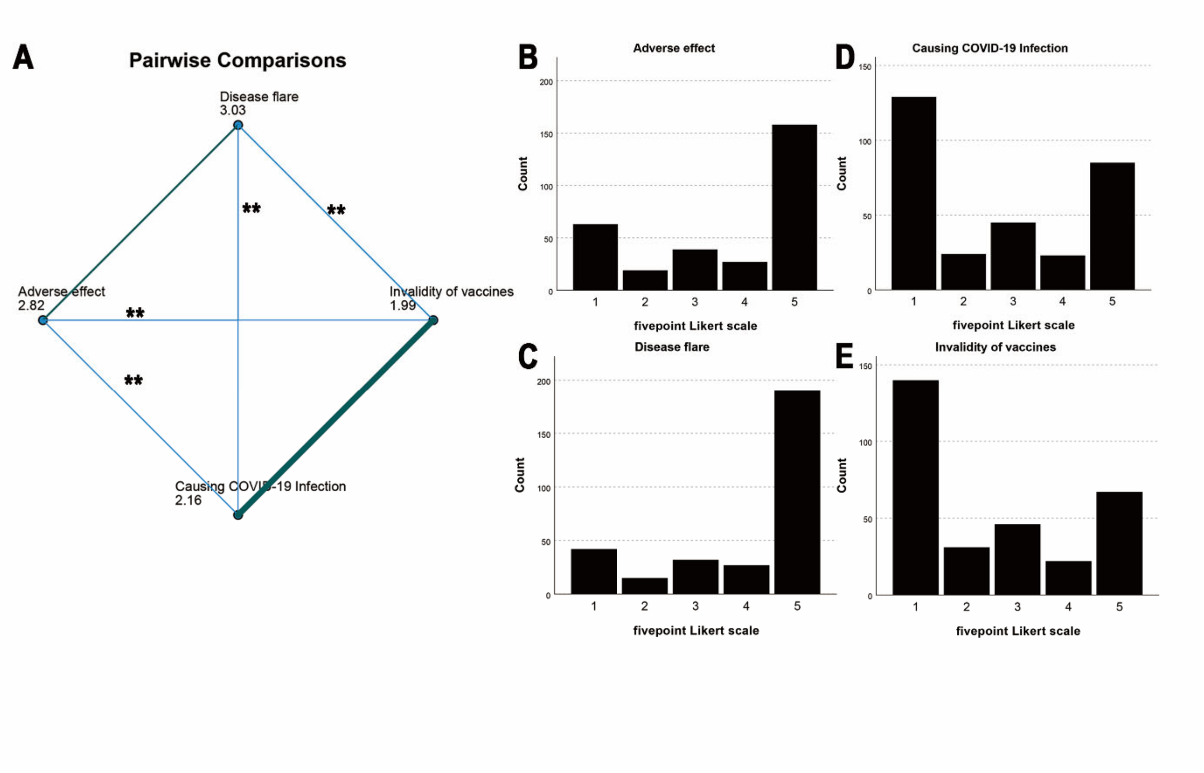

A: Pairwise Comparisons of the reasons for refusing vaccination. One asterisk (*) next to the connection indicates statistical difference at p <0.05, and two asterisks (**) indicate statistical difference at p <0.01. B-E: The count of five-point Likert scale of Adverse effect, Disease flare, Causing COVID-19 Infection and Invalidity of vaccines separately.

Figure S2. The Related-Samples Wilcoxon Signed Rank Test Summary of reasons for vaccination.

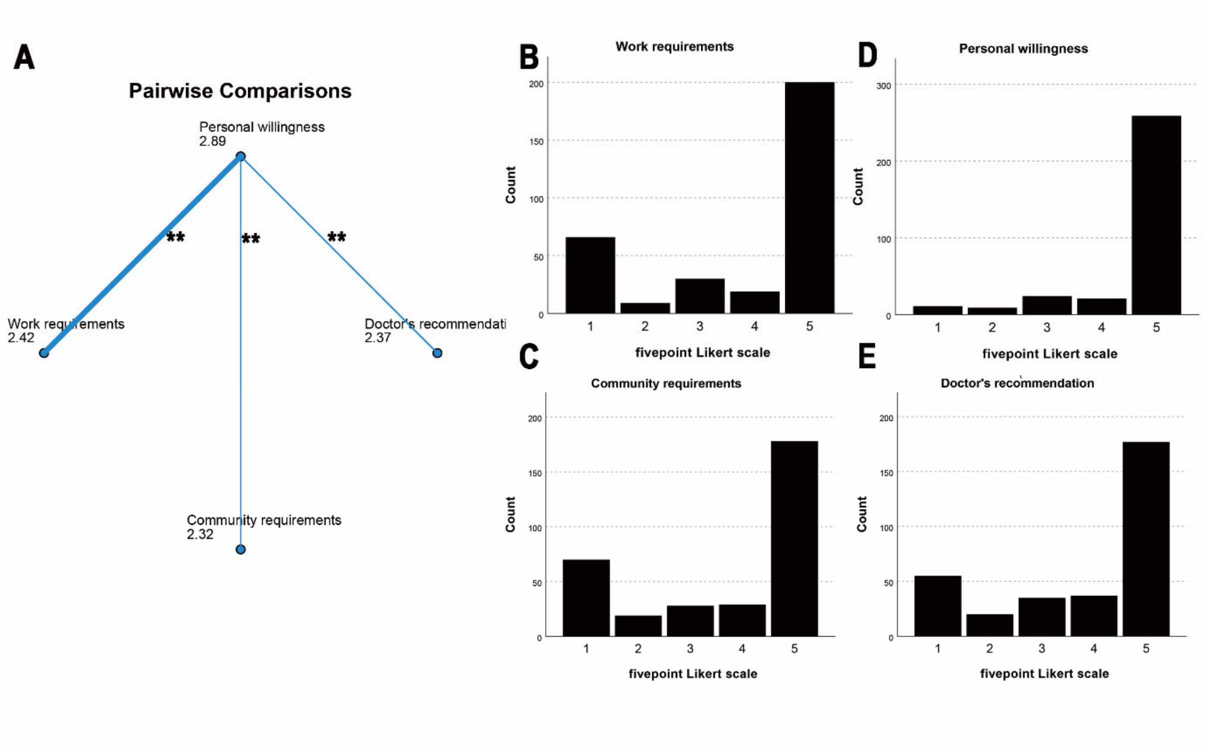

A: Pairwise Comparisons of the reasons for vaccination. One asterisk (*) next to the connection indicates statistical difference at $p < 0.05$, and two asterisks (**) indicate statistical difference at $p < 0.01$. B-E: The count of five-point Likert scale of Work requirements, Community requirements, Personal willingness and Doctor's recommendation separately.
